# Modeling cigarette smoking disparities between people with and without serious psychological distress in the US, 1997–2100

**DOI:** 10.1016/j.ypmed.2022.107385

**Published:** 2022-12-07

**Authors:** Qin Xi, Rafael Meza, Adam Leventhal, Jamie Tam

**Affiliations:** a Department of Public Health and Primary Care, University of Cambridge, Cambridge, UK; b Yale School of Public Health, 60 College St., New Haven, CT 06520, United States of America; c BC Cancer Research Institute, 675 West 10th Avenue, Vancouver, BC V5Z 1L3, Canada; d University of Southern California, School of Medicine, Soto Street Health Sciences Campus, Los Angeles, CA 90033, United States of America

**Keywords:** Smoking, Tobacco use, Health policy, Serious psychological distress, Health disparity, Mental health, Serious mental illness

## Abstract

Cigarette smoking rates are significantly higher among people with serious psychological distress (SPD) compared to the general population. US simulation models that project future smoking disparities by SPD status could inform policy interventions, but have not been developed.

We calibrated two compartmental models to the National Health Interview Survey 1997–2018 for populations with and without SPD, calculating smoking prevalence, mortality, and life-years lost by SPD status under different scenarios from 2023 to 2100.

Under the Status Quo, smoking prevalence among women with SPD falls from 27.0% in 2023 to 10.7% in 2100 (men: 30.1% to 12.2%). For women without SPD, it declines from 9.4% to 3.1% (men: 11.5% to 4.0%). The absolute difference in smoking prevalence between those with and without SPD decreases over time, whereas the relative smoking prevalence ratio increases. From 2023 to 2100, 609,000 premature smoking-attributable deaths would occur in the SPD population, with 8 million life-years lost. Under an ideal tobacco control scenario for people with SPD, in which all smokers quit in 2023 and no new smoking initiation occurs thereafter, up to 386,000 of these premature deaths could be averted with 4.9 million life-years gained. Preventing smoking initiation could avert up to 18% of these deaths, while improving smoking cessation could avert up to 82%.

Smoking-related disparities for people with SPD will persist unless a shift in tobacco control substantially improves cessation and prevents initiation in this subpopulation. Smoking disparities by SPD may widen in relative but narrow in absolute terms, so both perspectives should be evaluated.

## 1. Introduction

Addressing smoking disparities by mental health status remains a priority for public health decision-makers (Williams et al., 2013), as people with mental health conditions represent an increasing proportion of the remaining smoking population (Lawrence and Williams, 2016; Talati et al., 2016). In particular, people with serious psychological distress (SPD) comprise 3.2% of the US adult population, but 31.6% were current smokers in 2018 compared to 13.7% of the general population. As a result of their higher smoking rates, people with SPD have higher risk for cardiovascular disease, stroke, and cancer, and shorter life expectancies (McLachlan and Gale, 2018; Tam et al., 2016). While never smokers with SPD lose approximately 5 years of life expectancy compared to never smokers without SPD, current smokers with SPD lose approximately 15 years (Tam et al., 2016). People with SPD also have less success with quitting compared to those without SPD (Streck et al., 2020). Leading health organizations and government agencies are now convening to combat tobacco disparities for individuals with mental health conditions (The National Partnership on Behavioral Health and Tobacco Use - Healthier, n.d.). To quantify disparities and ensure progress towards health equity aims, tools to evaluate and monitor the long-term impact of current and potential policy and treatment interventions are needed.

Simulation models provide important information for decision-making to address health disparities (Smith et al., 2014; Diez Roux, 2011). Such models have been used extensively in tobacco control (Feirman et al., 2017), and are now being used to address tobacco-related health disparities (Le and Mendez, 2021; Institute USNC, 2017). Tam et al. developed a model examining smoking disparities for adults with a common mental health condition, major depression (MD), and the potential benefits of widespread cessation treatment for patients using mental health services (Tam et al., 2020; Tam et al., 2021a). No previous tobacco models have focused on the SPD population - a subgroup of the general population predisposed to experience severe mental illness and considerable impairment.

This study projects future smoking and related mortality among people with and without SPD over time, examining changes in disparities over time as well as the comparative benefits of intervention targeting initiation vs cessation strategies over time.

## 2. Methods

### 2.1. Data source

We use data from the National Health Interview Surveys (NHIS) 1997–2018, an annual cross-sectional nationally-representative household survey of the non-institutionalized civilian population (Davies et al., 2015). The NHIS has consistent measures for smoking and SPD from 1997 to 2018. Annually the number of adults survey ranged from 41,190 to 112,053, reflecting response rates that range from 62.7% to 80.4% (Tam et al., 2016). We exclude 2019-onwards NHIS surveys because of the absence of SPD measures.

### 2.2. Measures

Current smokers are those who currently smoke cigarettes every day or some days and who have smoked ≥100 cigarettes in their lifetime. Former smokers are those who have smoked ≥100 cigarettes in lifetime but do not currently smoke. Never smokers have smoked <100 cigarettes in their lifetime. Other combustible tobacco products such as cigars or pipe tobacco are not considered in the analysis.

SPD is a screening measure for identifying individuals with DSM-5 diagnoses, which can include bipolar disorder, schizophrenia, and severe depression or anxiety (Kessler et al., 2002; Kessler et al., 2003; Kessler et al., 2010; Furukawa et al., 2003). Psychological distress is assessed based on the Kessler six-item scale (Kessler et al., 2010), which asks about the frequency with which individuals experienced these symptoms in the past month (Kessler et al., 2010): nervousness, hopelessness, restlessness, sadness, like everything was an effort, and worthlessness. Each item is scored 0–4 based on the response “none of the time”, “a little of the time”, “some of the time”, “most of the time”, or “all of the time”. A person with a total score of ≥13 is considered to have serious psychological distress (SPD). Those with scores <13 are categorized as No SPD.

### 2.3. Modeling

We first develop and calibrate separate simulation models for the SPD and No SPD populations. Each model includes compartments for smoking status: current smoker, former smoker, and never smoker, with former smokers further distinguished by years since quitting (Appendix Fig. S1). Annual smoking initiation and cessation probabilities from the Cancer Intervention and Surveillance Modeling Network (CISNET) Lung consortium are based on the NHIS 1965–2018 (Jeon et al., 2018), and are used for the No SPD population model. For the SPD population model, initiation and cessation are re-calibrated to NHIS data on smoking among people with SPD; scaling factors by gender and age groups were calibrated to minimize the sum of squared differences between the model and survey data. Calibration assumed no smoking initiation after age 35, and no cessation prior to age 18. Cessation probabilities reflect permanent quitting among former smokers who last smoked ≥2 years ago, avoiding the need to model relapse. Given uncertainty about future smoking, we assume underlying initiation and cessation probabilities, and resulting differences between the SPD and no SPD populations, remain constant from 2018 onwards. This simplifies model projections and facilitates comparison across scenarios. Table 1 summarizes inputs and data sources.

We then make projections from 2023 to 2100 under three scenarios:

**Status Quo scenario** projecting a continuation of existing trends in which smoking initiation and cessation probabilities remain unchanged and fixed at the levels of year 2018. While these probabilities are kept constant, population smoking prevalence would still decrease into the future as older cohorts with higher initiation and lower cessation probabilities are replaced by younger cohorts with lower smoking initiation and higher cessation;

1. **No Initiation scenario** in which no new smoking initiation occurs from 2023 to 2100, but cessation rates remain unchanged over time; and
2. **Maximum Potential Reduction in Premature Mortality (MPRPM) scenario,** in which smokers across all ages immediately quit in 2024 and no new smoking initiation occurs from 2023 to 2100. This hypothetical scenario serves as a benchmark against which future treatment and policy interventions can be measured and accounts for the fact that even under maximal tobacco control conditions, there would still be some unavoidable smoking-attributable premature deaths due to former smokers’ increased mortality risk (Warner and Mendez, 2020).

Although it would be ideal to separately simulate a Complete Cessation scenario for comparison with the No Initiation scenario, this approach would be misleading. If all smokers permanently quit from 2023 to 2100, but never smokers can still continue to take up smoking, all current smokers from 2023 to 2100 would only remain a smoker for one year before quitting. Instead, we quantify the relative impact of cessation by taking the difference between MPRPM and No Initiation scenarios.

Outcomes include smoking prevalence, smoking-attributable deaths (SAD), and years of life lost (YLL). Smoking-attributable deaths (SAD) are calculated by:

$$SAD_{d}=\sum_{a,g}P\times (prev_{cs}\times (\mu_{cs,d}-\mu_{ns,d})+prev_{fs}\times (\mu_{fs,d}-\mu_{ns,d})\;)$$

where *P* is the size of the corresponding subpopulation for a given age (*a*), gender (*g*), or SPD status (*d*); *prev* is the prevalence of current or former smoking; and μ is the mortality rate of current, former, or never smokers. YLL is calculated by applying the remaining life expectancy (*e*) for a never smoker to each SAD: $YLL_{d}=\sum_{a},{\;}_{g}e_{ns},{\;}_{d}\times SAD_{d}$. Premature deaths averted and life years gained are calculated as the difference between the Status Quo and intervention scenarios.

To assess changes in the smoking disparity, we report both a relative and an absolute metric, as they can provide complementary information about the magnitude and patterns of disparities (Keppel et al., 2005; Identifying and Eliminating Tobacco-Related Disparities: Key Outcome Indicators for Evaluating Comprehensive Tobacco Control Programs—2022, 2021; King et al., 2012). As a relative measure of the disparity between SPD and no SPD populations, the smoking prevalence ratio can be calculated by dividing the prevalence of the former by that of the latter. As an absolute measure, the difference in smoking prevalence between both groups is reported (Keppel et al., 2005).

We do not consider the dynamics of psychological distress because its distribution in the population remains relatively unchanged (Tomitaka et al., 2019), SPD is not a diagnosable condition, and no data on transitions between SPD and No SPD status are available in the literature. Instead, two separate models were developed for the SPD and No SPD populations, based on the proportions of the population with and without SPD in 2018 (Table 1), and assuming that the distribution of psychological distress continues to remain stable over time (Tomitaka et al., 2019).

Analysis was conducted with R 4.1.0 (Team R, 2021) and the Bhat package in R (Luebeck and Meza, 2013). See Appendix for details.

## 3. Results

**Fig. 1.**
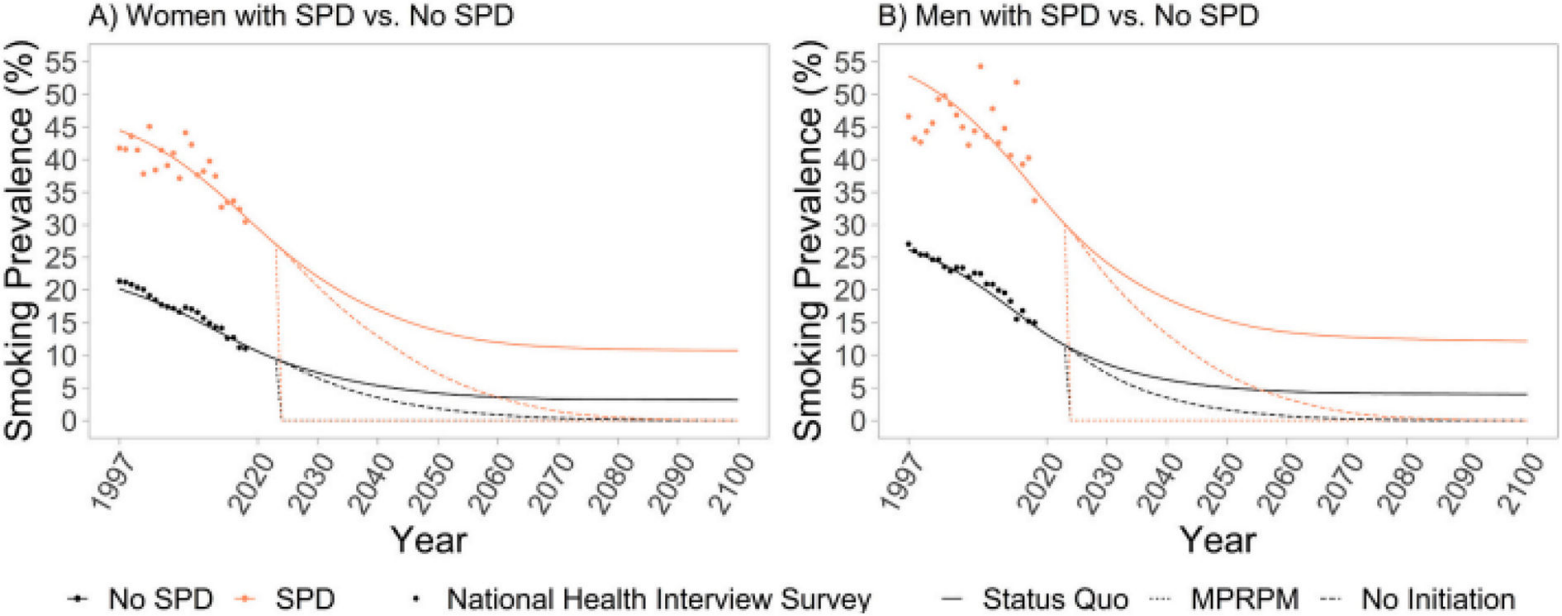
Smoking prevalence by SPD status under Status Quo, No Initiation, and MPRPM scenarios, 1997–2100. *Notes:* SPD = serious psychological distress. MPRPM = maximum potential reduction in premature mortality scenario in which all existing smokers immediately quit and no new smoking initiation occurs from 2024 to 2100.

Fig. 1 shows smoking prevalence model projections for men and women with and without SPD across the Status Quo, No-Initiation, and MPRPM scenarios. In the Status Quo scenario, smoking prevalence among women with SPD falls from 27.0% in 2023 to 10.7% by 2100. Smoking prevalence among women without SPD falls from 9.4% to 3.1% from 2023 to 2100. Similar declines are projected for males with SPD (30.1% to 12.2%) and without SPD (11.5% to 4.0%) (Table 2). Continued declines reflect the population replacement of older cohorts that have higher smoking rates with younger cohorts that have lower smoking rates.

Differences in smoking prevalence estimates between the Status Quo scenario and the No-Initiation scenario are initially minor but widen greatly over time (Fig. 1). In the No-Initiation scenario, smoking prevalence eventually drops to 0% for all groups. By 2040, relative changes of −60% and − 59% have occurred for women and men with SPD in 2040 compared to 2023. For those without SPD, the relative changes in prevalence were − 67% and − 65% for adult women and men in 2040. Under the MPRPM scenario starting in 2024, smoking prevalence for all groups immediately drops to 0%.

Under the Status Quo scenario, women’s smoking-attributable fraction (SAF) of deaths, or the proportion of all fatalities that can be attributed to smoking, drop from 12.7% in 2023 to 3.0% in 2100 among those with SPD, and from 7.1% to 0.6% among those without SPD (See Appendix Table S2). For men with SPD, the proportion declines from 17.2% in 2023 to 5.2% in 2100, compared to 15.2% to 1.5% in men without SPD. From 2023 to 2100, cumulative smoking-related mortality among US adults with SPD is 609,000 premature deaths, resulting in a total loss of 8 million life-years. Mortality estimates are rounded to the nearest thousand in the text, while life-years are presented in millions.

Under the No-Initiation scenario, from 2023 to 2100, the cumulative number of total smoking-attributed deaths among adults with SPD is 539,000, with 6.8 million life-years lost. The smoking-attributable fraction of all deaths declines from 12.7% to 0.3% in 2100 for women with SPD, and from 7.1% to 0.1% for women without SPD; for men with SPD it declines from 17.2% to 0.3% and for men without, from 15.2% to 0%.

In the MPRPM scenario, from 2023 to 2100, total smoking-attributed mortality among US adults with SPD is 224,000 premature deaths, and 3.1 million life-years are lost. By 2100, the MPRPM scenario averts 63.3% of all SADs and 61.7% of life-years lost among adults with SPD. The remaining proportion represent deaths that cannot be averted with further intervention, due to former smokers’ unavoidable increased mortality risk, even after quitting. Additional results provided in Appendix Table S2.

Table 3 shows the number of premature deaths averted and life-years gained by scenario, and assesses the maximum possible contribution of smoking prevention efforts vs smoking cessation efforts to the MPRPM scenario. Under the MPRPM scenario, the immediate public health benefits are 100% attributed to smoking cessation and 0% to prevention of smoking initiation for both the SPD and No SPD populations. Health gains for the SPD population under the No-Initiation scenario gradually achieves 18% of deaths averted and 24% of life-years gained in the MPRPM scenario by 2100. However long-term health gains are dominated by improvements to smoking cessation, comprising the remaining 82% and 76% of premature deaths averted and life-years gained for the SPD population.

## 4. Discussion

This is the first modeling study of smoking disparities for US adults with and without SPD. We project that nearly 609,000 premature deaths attributable to smoking will occur among the ~3% of US adults with SPD from 2023 to 2100 if current trends continue. This translates to a projected 8 million life-years lost for adults with SPD. By 2100, the proportion of deaths attributable to smoking in the SPD population will be several times as large as that of the No SPD population (3% vs 0.6% for women and 5.2% vs. 1.5% for men), and SPD smoking prevalence will be more than three times that of the No SPD population (10.7% vs 3.1% for women and 12.2% vs 4% for men). While smoking is expected to decline in absolute terms in all groups over this time period, disparities will persist, and even increase in relative terms, in the long-term. Eliminating disparities can become more challenging when the benchmark for equity represents a moving target; that is, as long as people without SPD reduce their smoking at a faster rate than people with SPD, the more difficult it will be for the latter group to catch up.

Whether smoking-related disparities will improve or worsen over time depends on what type of metric is used (Identifying and Eliminating Tobacco-Related Disparities: Key Outcome Indicators for Evaluating Comprehensive Tobacco Control Programs—2022, 2021). When using a *relative* burden metric for disparities, our modeling suggests that the smoking prevalence ratio between people with and without SPD will continue to increase over time. Our findings are consistent with that of Tam et al., which found that the smoking prevalence ratio between those with current major depression and those without a history of major depression will increase without intervention (Tam et al., 2020). When using a strictly *absolute* burden metric for disparities based on the raw difference in the percentage of smoking between people with and without SPD, the gap between both groups will narrow over time, as overall prevalence becomes closer to 0. Since the SPD group has a higher smoking prevalence to begin with, they also have more room for improvement compared to the No SPD group, for whom smoking has declined so much that it is approaching a plateau.

The different conclusions across the relative and absolute burden of disparities estimates highlights the importance of evaluating how trends in disparities are reported, as disparities might be narrowing or widening dependng on whether an absolute or relative metric is used (Keppel et al., 2005). Such distinctions are critical for policy makers determining whether current disparities trends are unacceptable. Relative metrics value fairness as a principle and take an explicit equity perspective, but they do not account for overall population health trends (i.e. relative differences between groups can decrease over time, even if smoking rates were increasing for the overall population). If the public health objective is to ensure that both advantaged and disadvantaged groups share equally in the harms of smoking (or the benefits of interventions), then relative metrics are appropriate. Absolute metrics account for overall population health but are not assessing fairness per se; that is, as smoking prevalence declines for SPD and no SPD populations, fewer people in absolute numbers are harmed by smoking, even if the harm remains unequally distributed. Future estimates should adhere to CDC recommendations and consider both metrics (Identifying and Eliminating Tobacco-Related Disparities: Key Outcome Indicators for Evaluating Comprehensive Tobacco Control Programs—2022, 2021).

We evaluated hypothetical scenarios that assess the potential short- and long-term impact of tobacco interventions targeting smoking initiation vs. cessation for the SPD and non-SPD populations. Although MPRPM is not a realistic projection, it is an aspirational goal against which all future interventions can be measured, and provides an ideal tobacco control scenario in which both elimination of smoking initiation and population-wide immediate smoking cessation are maximally achieved (Tam et al., 2021a). The MPRPM scenario is also a more appropriate benchmark for evaluation: Analyses that only compare intervention scenarios with the Status Quo ignore the fact that some deaths (former smokers’) cannot be prevented with intervention. Not only does the MPRPM scenario as a reference point evaluate the magnitude of possible public health impact, it also reveals the timing of when benefits are achieved. When compared with the MPRPM, benefits from the No Initiation scenario appear in later years, after young people who were diverted away from smoking reach older ages when resulting reductions to mortality risk are finally observable. By the end of this century, initiation efforts could achieve 18% of all possible mortality reductions at most in the SPD population. The remaining 82% could be averted only through cessation efforts. Thus improvements to smoking cessation for people with SPD would immediately reduce harms and achieve a higher percentage of the benefits of the MPRPM scenario in the short-run, but in the long-run, preventing smoking initiation among young people with signs of mental illness would prevent disease and disability in later life.

### 4.1. Strengths and limitations

Our study is strengthened by the use of the NHIS, which is the most historically comprehensive data source on smoking and SPD in the US. Another strength is use of age-specific mortality and life expectancy data that are specific to both SPD and smoking status (Tam et al., 2016). The CISNET initiation and cessation probabilities used in the models are widely used in tobacco simulation modeling, (Jeon et al., 2018; Tam et al., 2018; Tam et al., 2021b) and were recalibrated to match observed smoking prevalence for the SPD population.

This study should be considered in light of several limitations. First, we assumed that people maintain the same mental health status across their lifetime. Second, this study uses compartmental models, and not individual-based models; therefore the study findings reflect changes for the population on aggregate, rather than the experience of individuals. Third, after 2018, the NHIS removed questions about SPD, preventing our model from being calibrated to more recent data. This impacts how we evaluate trends going forward and speaks to the importance of having consistent measures for mental health in national surveys. Future research will need to develop methods to make other measurements of mental distress comparable with previous data. Fourth, we make the conservative assumption that differences in underlying smoking initiation and cessation probabilities between people with and without SPD remain constant beyond 2018. These are kept constant, but smoking prevalence continues to decline under the Status Quo scenario as younger cohorts replace older cohorts. If differences between the two groups have increased since 2018, our results would underestimate absolute and relative changes to disparities over time. However, our findings regarding the relative short-term and long-term impact of interventions targetting initiation vs. cessation would remain the same – cessation would make up the majority of impact in the short term, while the impact of initiation would emerge in the long term. Fifth, this modeling study does not account for the disruptive impact of COVID-19 and how that could impact future trends. Future patterns could also be influenced by global conflict, improvements to medicine and healthcare access, or the onset of other pandemics.

The models do not consider changes to SPD status at the individual or population levels. We assume the proportion of the population with SPD does not change over time, consistent with NHIS data, and that people maintain the same mental health status across their lifetime. Future studies could look at the interactions between smoking and SPD, or generate parameters specific to the epidemiology of SPD that could be used for modeling purposes. The interplay between smoking and SPD could be explored in subsequent research (Zvolensky et al., 2018). This study does not address non-cigarette tobacco products. Beyond cigarettes, rates of e-cigarette use, and dual use are higher among adults with SPD than those without SPD (McLachlan and Gale, 2018). Future research should evaluate trends in e-cigarette use among people with mental illness and how this impacts their smoking.

### 4.2. Implications

Smoking disparities between people with and without SPD will persist and increase under some metrics unless bold action is taken. National treatment recommendations to offer smokers with psychological distress cessation counseling and pharmacotherapy should be followed more rigorously, with extra care to support long-term abstinence given their higher potential for failed quit attempts and early relapse back to smoking (Williams et al., 2011). Other interventions, including contingency management (Cahill et al., 2015) and use of e-cigarettes to support cigarette smoking cessation (Hartmann-Boyce et al., 2016), may also increase quit rates but require further evaluation in this population. Though largely absent from the tobacco control armamentarium, targeted interventions to prevent smoking initiation among young people with psychological distress should be pursued, similar to those countermarketing campaigns that have already being employed to prevent vaping initiation (Feirman et al., 2016).

Future federal actions could dramatically improve health for the SPD population. If the US Food and Drug Administration (FDA) reduces nicotine levels in cigarettes to minimally addictive levels, clinical trial data suggests that this could facilitate quitting among people with psychiatric conditions (Higgins et al., 2020). The FDA recently announced plans to prohibit menthol as a characterizing flavor in cigarettes (Genetic Testing, 2022). Since people with psychological distress, especially young adults, are more likely to smoke menthol cigarettes (Hickman et al., 2014; Brunette et al., 2019; Young-Wolff et al., 2015), such a regulation could also reduce their smoking. Because they would reduce the appeal and addictiveness of cigarettes, both the proposed ban on menthol flavors and plans to reduce nicotine levels would logically prevent smoking initiation among young people with mental health conditions who may try smoking. The CDC campaign *Tips from Former Smokers* included messaging targetted for people with mental health conditions (Tips from Former Smokers, 2022); similar public education campaigns could be used to target young people with mental health conditions and emphasize that smoking is not a healthy or effective way to manage their symptoms. Federal regulation, combined with increased access to effective smoking cessation interventions (Gilbody et al., 2019; Simonavicius et al., 2017) and other policies (e.g., raising cigarette taxes, implementing mass media messaging in settings overrepresented by people with SPD) are needed to maximize impact.

## 5. Conclusions

The SPD model used in this study is one of only two that evaluates smoking among people with mental conditions in the US (Tam et al., 2020). Such models can facilitate surveillance of tobacco use in high priority populations and inform decision-making around what types of policy and treatment interventions to implement and how they should be leveraged to maximize health gains. Changes to the NHIS that remove long-standing mental health measures such as SPD present challenges for population health surveillance and modeling efforts. Future research will need to rely on alternative data sources to assess trends in tobacco product use among people with SPD.

## Supplementary Material

Supplement

## Figures and Tables

**Table 1:** Inputs and data sources for modeling smoking by SPD status.

Inputs	Description and data source					
Births and population size	Births to simulated population based on US Census Bureau population estimates Population sizes scaled according to proportion of the population with and without SPD by gender and age group in the NHIS 1997–2018:					
	SPD	18–34	35–54	55–64	65–74	75+
	Men	2.1%	2.9%	3.2%	1.9%	1.9%
	Women	3.3%	4.4%	4.6%	2.9%	2.8%
	The no SPD population is the complement to the SPD distribution (no SPD % = 1 – SPD %).					
Smoking initiation	CISNET smoking initiation probabilities by age, gender, and birth cohort-specific generated using the NHIS 1965–2018 (Jeon et al., 2018). CISNET initiation inputs are calibrated to smoking distribution by SPD, age, and gender in the NHIS 1997–2018 using age group-specific scaling factors.					
Smoking cessation	CISNET smoking cessation probabilities by age, gender, and birth cohort-specific generated using the NHIS 1965–2018 (Jeon et al., 2018). Based on former smokers who quit at least two years ago in the NHIS and therefore reflects permanent quitting with no relapse back to smoking. CISNET cessation inputs are calibrated to smoking distribution by SPD, age, and gender in the NHIS 1997–2018 using age group-specific scaling factors.					
All-cause mortality	Individuals exit the model by death or at age 99. US all-cause mortality rates from the human mortality Database (Human Mortality Database, 2017) are used as underlying rates multiplied by mortality Hazard ratios for current and former smokers (reference Is never smokers) and SPD (reference is non-SPD). Hazard ratios based on a previous analysis of the NHIS-NDI mortality follow-up data (Tam et al., 2016). Former smoker mortality is distinguished by years since quitting. The death rates for former smokers in each compartment by age and year are derived from previous literature (Tam et al., 2021c). An exponential interpolation is used to derive the relative death rate for former smokers compared to current smokers. Thus, the death rate of former smokers decreases with the number of years since quit.					
Life expectancy	Lifetables by age, gender, smoking status, and SPD status generated using the human mortality database protocol and previously reported estimates (Tam et al., 2016; Human Mortality Database, 2017).					

*Notes:* SPD = serious psychological distress.

**Table 2:** Smoking disparity measures for adults with and without serious psychological distress, Status Quo scenario, 2023–2100.

	Year	2023	2040	2060	2080	2100	Change from 2023 to 2100
Women	SPD smoking prevalence	27.0%	17.0%	12.0%	10.9%	10.7%	−60%
	Non-SPD smoking prevalence	9.4%	5.4%	3.6%	3.2%	3.1%	−67%
	Absolute difference	17.6%	11.6%	8.4%	7.7%	7.6%	
	Relative difference	1.9	2.1	2.3	2.4	2.5	
	Prevalence ratio	2.9	3.1	3.3	3.4	3.5	
Men	SPD smoking prevalence	30.1%	18.7%	13.6%	12.5%	12.2%	−59%
	Non-SPD smoking prevalence	11.5%	6.3%	4.5%	4.1%	4.0%	−65%
	Absolute difference	18.2%	12.0%	9.6%	8.4%	7.6%	
	Relative difference	1.6	2.0	2.0	2.0	2.1	
	Prevalence ratio	2.6	3.0	3.0	3.0	3.1	

*Notes:* Summary table of model projections for men and women with and without past year serious psychological distress (SPD). Current smoking is defined as smoking within the past year.

**Table 3:** Projected population health gains under MPRPM and No-Initiation scenarios each, relative to the Status Quo, 2023–2100.

SPD	Cumulative premature deaths averted					Cumulative life-years gained				
Year	No-initiation scenario	MPRPM scenario	Difference (complete cessation)	No-initiation as % of MPRPM	Complete cessation as % of MPRPM	No-initiation scenario	MPRPM scenario	Difference (complete cessation)	No-initiation as % of MPRPM	Complete cessation as % of MPRPM
2030	0	22,209	22,209	0.00%	100.00%	0	300,086	300,086	0.00%	100.00%
2040	1	79,510	79,509	0.00%	100.00%	11	1,151,416	1,151,405	0.00%	100.00%
2050	20	152,795	152,775	0.01%	99.99%	371	2,121,556	2,121,185	0.02%	99.98%
2060	843	221,366	220,523	0.38%	99.62%	19,713	2,949,742	2,930,029	0.67%	99.33%
2070	6531	275,751	269,220	2.37%	97.63%	140,960	3,584,136	3,443,176	3.93%	96.07%
2080	21,484	318,331	296,847	6.75%	93.25%	432,082	4,088,688	3,656,606	10.57%	89.43%
2090	43,066	353,509	310,443	12.18%	87.82%	797,883	4,527,661	3,729,778	17.62%	82.38%
2100	69,868	385,513	315,645	18.12%	81.88%	1,178,656	4,927,790	3,749,134	23.92%	76.08%
No										
SPD	Cumulative premature deaths averted					Cumulative life-years gained				
Year	No-initiation scenario	MPRPM scenario	Difference (complete cessation)	No-initiation as % of MPRPM	Complete cessation as % of MPRPM	No-initiation scenario	MPRPM scenario	Difference (complete cessation)	No-initiation as % of MPRPM	Complete cessation as % of MPRPM
2030	6	255,118	255,112	0.00%	100.00%	90	3,292,653	3,292,563	0.00%	100.00%
2040	629	982,226	981,597	0.06%	99.94%	18,011	13,609,484	13,591,473	0.13%	99.87%
2050	7776	1,978,991	1,971,215	0.39%	99.61%	226,504	26,136,811	25,910,307	0.87%	99.13%
2060	45,727	2,933,944	2,888,217	1.56%	98.44%	1,355,326	37,711,668	36,356,342	3.59%	96.41%
2070	143,327	3,622,757	3,479,430	3.96%	96.04%	3,993,275	46,616,370	42,623,095	8.57%	91.43%
2080	318,376	4,105,117	3,786,741	7.76%	92.24%	8,096,203	53,373,888	45,277,685	15.17%	84.83%
2090	558,858	4,478,897	3,920,039	12.48%	87.52%	12,941,540	59,091,987	46,150,447	21.90%	78.10%
2100	848,085	4,810,738	3,962,653	17.63%	82.37%	17,882,012	64,232,070	46,350,058	27.84%	72.16%

*Notes:* No Initiation scenario = No new smoking initiation occurs from 2024 to 2100, while smoking cessation patterns remain constant over time; MPRPM scenario = Maximum Potential Reduction in Premature Mortality scenario in which all existing smokers immediately quit and no new smoking initiation occurs from 2023 to 2100. Complete cessation = the net contribution of smoking cessation to the MPRPM taken as the difference between the MPRPM and No Initiation scenarios.

## Data Availability

Data will be made available on request.
