# Supplementary material for: Modeling cigarette smoking disparities between people with and without serious psychological distress in the US, 1997–2100
Source: Prev Med. Author manuscript; Available in PMC 2023 Mar 8. (PMC9994605; doi:10.1016/j.ypmed.2022.107385)
Supplement: Supplement [file NIHMS1875075-supplement-Supplement.pdf]

Appendix

Figure S1. Model diagram of smoking among people with and without SPD

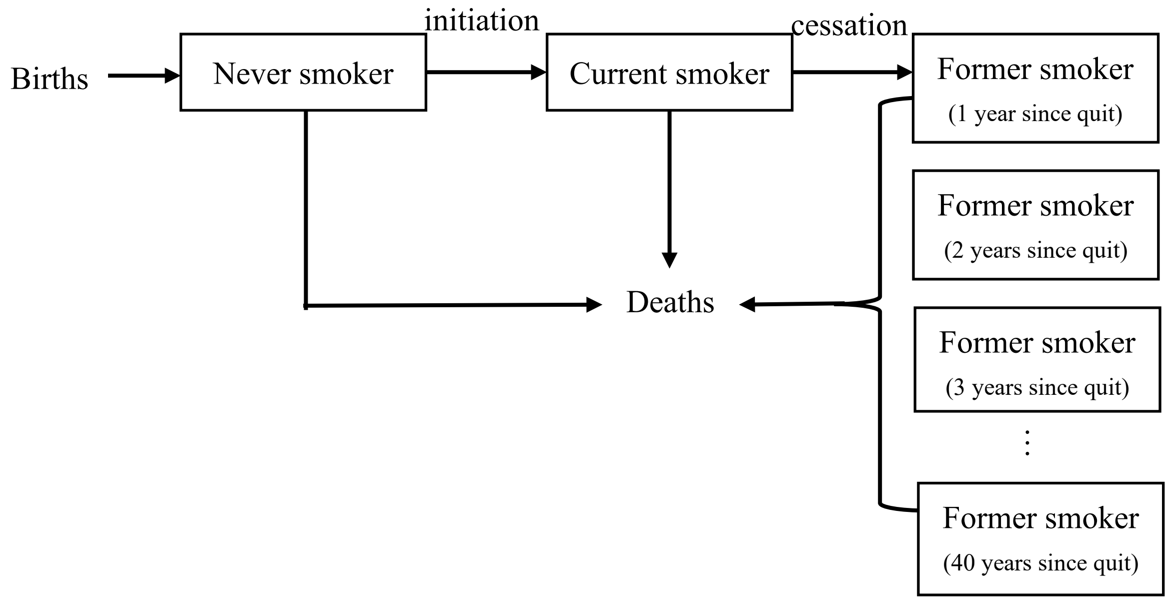

Notes: SPD = serious psychological distress. Each of the SPD and No SPD populations are separately simulated at birth as never smokers, who may transition to current smoking and subsequently quit to become former smokers.

Figure S2. Smoking prevalence among adults with and without SPD, NHIS and model estimates, 1997-2018.

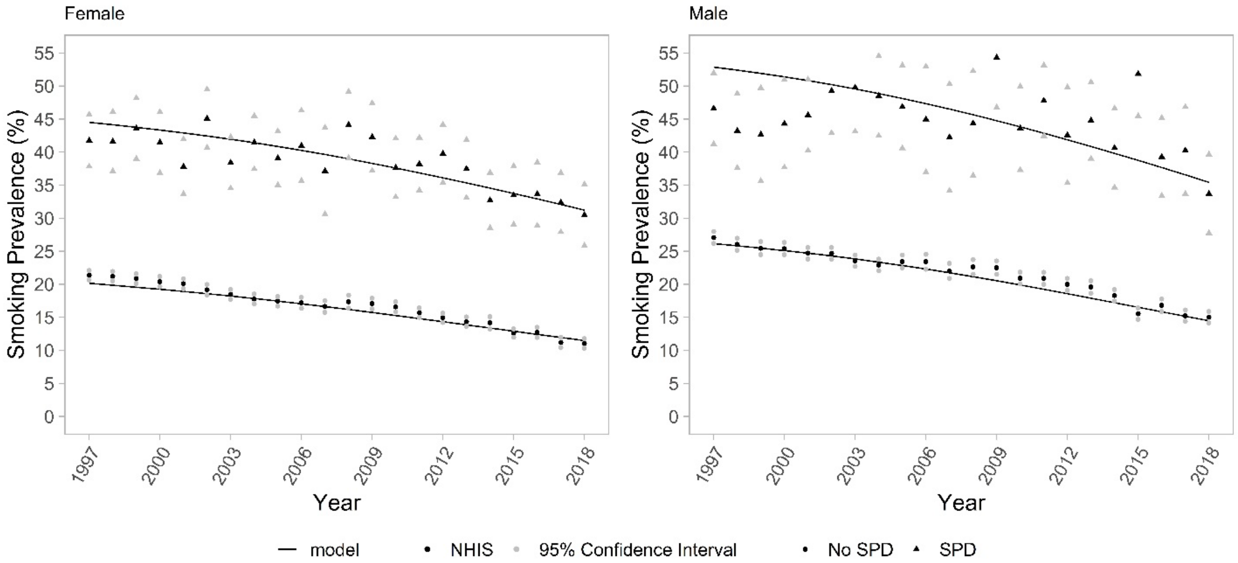

Both SPD and No SPD model estimates of smoking prevalence when applied to 1997-2018 year correspond well with the observed prevalences in NHIS 1997-2018 data (Appendix Figure S2), though survey estimates have wider confidence intervals due to smaller sample sizes for people with SPD. From 1997-2018, smoking prevalence among women with SPD declined from 44.5% to 31.2% in the model, compared to 41.7% (95% CI: 37.8 – 45.7) and 30.5% (95%CI: 25.8 – 35.1) in the NHIS. For men with SPD, prevalence decreased from 52.9% to 35.4% in the model and from 46.6% (95%CI: 41.2 – 52.0) to 33.7% (95% CI: 27.7 – 39.6) in the NHIS. Over this same time period, women without SPD experienced a decline of 20.2% to 11.5% in the model and 21.4% (95% CI: 20.6 – 22.1) to 11.0% (95% CI: 10.3 – 11.7) in the NHIS; for men without SPD, it decreased from 26.2% to 14.5% in the model and from 27.1% (95% CI: 26.1 – 28.0) to 15.0% (95% CI: 14.1 – 15.9) in the NHIS. According to NHIS data, the smoking prevalence ratio between women with and without SPD increased from 1.9 to 2.8, and for men with and without SPD, from 1.7 to 2.2 (Appendix Table S1).

Table S1. Change in smoking prevalence among adults with and without SPD, NHIS 1997-2018.

	**Year**	**1997**	**2018**	**Change from 1997-2018**
**Women**	SPD smoking prevalence (95% CI)	41.7% (37.8, 45.7)	30.5% (25.8, 35.1)	-11.2% (-12.0, -10.6)
	No or low PD - smoking prevalence (95% CI)	21.4% (20.6, 22.1)	11.0% (10.3, 11.7)	-10.4% (-10.3, -10.4)
	Absolute difference	20.3% (17.2, 23.6)	19.5% (15.5, 23.4)	-0.8% (-1.7, -0.2)
	Prevalence ratio	1.9 (1.8, 2.1)	2.8 (2.5, 3)	1.1 (1.2, 1)
**Men**	SPD smoking prevalence (95% CI)	46.6% (41.2, 52.0)	33.7% (27.7, 39.6)	-12.9% (-13.5, -12.4)
	No or low PD - smoking prevalence (95% CI)	27.1% (26.1, 28.0)	15.0% (14.1, 15.9)	-12.1% (-12.0, -12.1)
	Absolute difference	19.5% (15.1, 24.0)	18.7% (13.6, 23.7)	-0.8% (-1.5, -0.3)
	Prevalence ratio	1.7 (1.6, 1.9)	2.2 (2.0, 2.5)	1.1 (1.1, 1)

Notes: PD = psychological distress. PD is assessed using the Kessler six-item (K6) scale which assesses the frequency with which individuals experienced symptoms of distress within the past month. Serious Psychological Distress (SPD) = K6 score ≥ 13. Moderate PD = K6 score 5-12. No or Low PD = K6 score 0-4.

Table S2. Smoking-attributed mortality and life-years lost among US adults with and without SPD by scenario, 2023-2100

Women	SPD				No SPD			
	Status Quo scenario		No Initiation scenario	MPRPM scenario	Status Quo scenario		No Initiation scenario	MPRPM scenario
	2023	2100	2100	2100	2023	2100	2100	2100
Smoking prevalence (%)	27	10.7	0	0	9.4	3.1	0	0
Proportion of all deaths attributed to smoking (%)	12.7	3	0.3	0	7.1	0.6	0.1	0
Annual number of deaths	8,023	1,339	131	0	109,378	8,928	960	0
Cumulative number of deaths	8,023	281,289	254,240	74,045	109,378	3,228,232	3,074,264	1,184,334
Annual years of life lost	125,414	16,242	310	0	1,652,274	109,281	2,562	0
Cumulative years of life lost	125,414	3,661,340	3,188,558	1,028,402	1,652,274	40,471,813	37,691,352	16,366,029
**Men**	**SPD**				**No SPD**			
	**Status Quo scenario**		**No Initiation scenario**	**MPRPM scenario**	**Status Quo scenario**		**No Initiation scenario**	**MPRPM scenario**
	**2023**	**2100**	**2100**	**2100**	**2023**	**2100**	**2100**	**2100**
Smoking prevalence (%)	30.1	12.2	0	0	11.5	4	0	0
Proportion of all deaths attributed to smoking (%)	17.2	5.2	0.3	0	15.2	1.5	0	0
Annual number of deaths	9,362	1,804	103	0	240,667	23,404	759	0
Cumulative number of deaths	9,362	328,022	285,203	149,753	240,667	6,836,414	6,142,297	4,069,574
Annual years of life lost	140,329	22,088	205	0	4,100,286	380,157	1,944	0
Cumulative years of life lost	140,329	4,321,973	3,616,099	2,027,121	4,100,286	104,281,105	89,179,554	64,154,819

Notes: SPD = Serious psychological distress; No Initiation scenario = No new smoking initiation occurs from 2024-2100, while smoking cessation patterns remain constant over time; MPRPM scenario = Maximum Potential Reduction in Premature Mortality scenario in which all existing smokers immediately quit and no new smoking initiation occurs from 2023-2100
